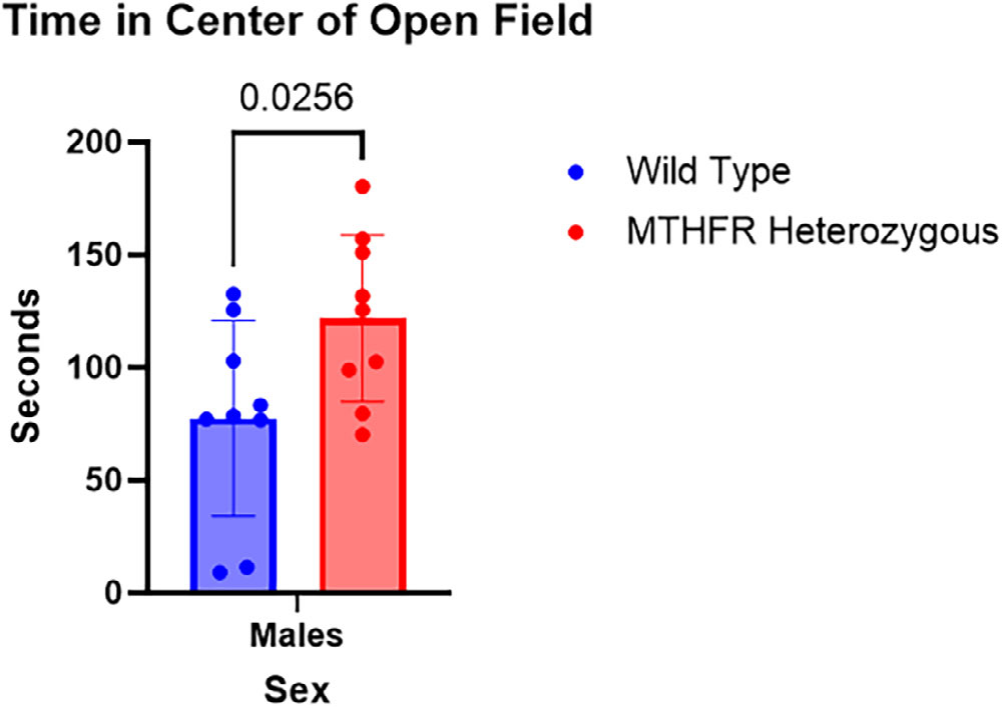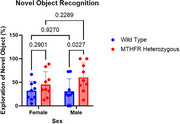# Sex‐Specific Differences in Cognition of Mthfr Deficient Mice

**DOI:** 10.1002/alz70858_104546

**Published:** 2025-12-25

**Authors:** Gregory W Hall, Meenakshi Umar, Gregory J Bix

**Affiliations:** ^1^ Department of Neurosurgery, Clinical Neuroscience Research Center, Tulane University School of Medicine, New Orleans, LA, USA; ^2^ Tulane University, New Orleans, LA, USA; ^3^ Tulane University School of Medicine, New Orleans, LA, USA; ^4^ Tulane Brain Institute, Tulane University, New Orleans, LA, USA; ^5^ Department of Neurology, Tulane University School of Medicine, New Orleans, LA, USA

## Abstract

**Background:**

Methylenetetrahydrofolate reductase (Mthfr) catalyzes the major circulating form of folate. Common Mthfr polymorphisms increase the risk of various neurological disorders. Moreover, heterozygous knockout (Mthfr +/‐) mice show diminished blood‐brain‐barrier integrity, reduced pericytes, and reduced vascular density. However, data on behavioral performance of Mthfr +/‐ mice is limited. Behavioral data can measure cognitive impairment. We hypothesize that Mthfr+/‐ mice display cognitive deficits as compared to WT mice in behavioral tests. Therefore, we used Mthfr +/‐ mice (to model Mthfr polymorphisms) and aged matched WT litter mates to investigate the effect of Mthfr deficiency in cognitive performance.

**Method:**

11‐13 months old male and female Mthfr heterozygous knockout (Mthfr+/‐) mice and aged‐matched litter mate WT mice were evaluated (*n* = 36). Open field and novel object recognition tests measured cognition through categories associated with cognitive senescence: anxiety, exploratory behavior, recognition memory, and learning capacity.

**Result:**

While overall differences between the two groups were not significant, sex stratification revealed significant differences between Mthfr +/‐ males and male WT counterparts for both cognitive tests. Mthfr +/‐ male mice averaged 121.889 seconds in the open field's center, while male WT mice averaged 77.444 seconds (*p* = 0.0256). Additionally, Mthfr +/‐ males reduced speed in the open field's center when compared to their WT counterparts (*p* = 0.0093). For novel object recognition test, Mthfr +/‐ males averaged an exploratory ratio of 60.591% while WT males averaged 30.884% (*p* = 0.0227). Mean speed and distance traveled were comparable for Mthfr +/‐males and WT males in both cognitive tests. Female Mthfr +/‐ mice showed no significant differences from WT counterparts in these behavior tests.

**Conclusion:**

Mthfr deficiency causes previously uncategorized sex‐dependent (male) effects on cognition (reduced anxiety, enhanced exploration, enhanced recognition memory, and enhanced learning capacity). Thus, the efficacy of Mthfr as a target for therapeutic interventions for ameliorating cognitive deficits may vary depending on sex. Furthermore, Mthfr polymorphisms may ameliorate age associated cognitive decline in males. Future investigations should include additional cognitive assessments such as passive avoidance tests to further categorize potential sex‐dependent differences between Mthfr +/‐ and WT mice as well as consider other criteria including age.